# Misfolding of CasBrE SU is reversed by interactions with 4070A Env: implications for gammaretroviral neuropathogenesis

**DOI:** 10.1186/1742-4690-7-93

**Published:** 2010-11-05

**Authors:** Ying Li, William P Lynch

**Affiliations:** 1 Department of Integrative Medical Sciences, Northeastern Ohio Universities College of Medicine, 4209 State Route 44, Rootstown, Ohio 44272, USA; 2 School of Biomedical Sciences, Kent State University, Kent, Ohio, USA; 3 Departments of Neurology and Neuroscience Johns Hopkins University School of Medicine Baltimore, MD 21205, USA

## Abstract

**Background:**

CasBrE is a neurovirulent murine leukemia virus (MLV) capable of inducing paralytic disease with associated spongiform neurodegeneration. The neurovirulence of this virus has been genetically mapped to the surface expressed subunit (SU) of the *env *gene. However, CasBrE SU synthesized in the absence of the transmembrane subunit (TM) does not retain ecotropic receptor binding activity, indicating that folding of the receptor binding domain (RBD) requires this domain. Using a neural stem cell (NSC) based viral *trans *complementation approach to examine whether misfolded CasBrE SU retained neurovirulence, we observed CasBrE SU interaction with the "non-neurovirulent" amphotropic helper virus, 4070A which restored functional activity of CasBrE SU.

**Results:**

Herein, we show that infection of NSCs expressing CasBrE SU with 4070A (CasES+4070A-NSCs) resulted in the redistribution of CasBrE SU from a strictly secreted product to include retention on the plasma membrane. Cell surface cross-linking analysis suggested that CasBrE SU membrane localization was due to interactions with 4070A Env. Viral particles produced from CasES+4070A-NSCS contained both CasBrE and 4070A gp70 Env proteins. These particles displayed ecotropic receptor-mediated infection, but were still 100-fold less efficient than CasE+4070A-NSC virus. Infectious center analysis showed CasBrE SU ecotropic transduction efficiencies approaching those of NSCs expressing full length CasBrE Env (CasE; SU+TM). In addition, CasBrE SU-4070A Env interactions resulted in robust ecotropic superinfection interference indicating near native intracellular SU interaction with its receptor, mCAT-1.

**Conclusions:**

In this report we provided evidence that 4070A Env and CasBrE SU physically interact within NSCs leading to CasBrE SU retention on the plasma membrane, incorporation into viral particles, restoration of mCAT-1 binding, and capacity for initiation of TM-mediated fusion events. Thus, heterotropic Env-SU interactions facilitates CasBrE SU folding events that restore Env activity. These findings are consistent with the idea that one protein conformation acts as a folding scaffold or nucleus for a second protein of similar primary structure, a process reminiscent of prion formation. The implication is that template-based protein folding may represent an inherent feature of neuropathogenic proteins that extends to retroviral Envs.

## Introduction

Certain MLVs are capable of causing severe progressive non-inflammatory spongiform motor neuron disease when inoculated into susceptible neonatal mice. The prototypic virus of this class, referred to as CasBrE, was first isolated from wild mice and shown to cause a paralytic wasting disease with low incidence and a long incubation period, highly reminiscent of certain prion diseases and amyotrophic lateral sclerosis (ALS) [1-3]. Genetic mapping studies have indicated that the primary viral neurovirulence determinants reside within the viral *env *gene, specifically to within the region encoding the SU subunit (cf., [4-10]). As a result, it has been speculated that CNS expression of neurovirulent MLV Env protein alone might be sufficient for inducing neurodegeneration [4]. Attempts to address this issue through the generation of transgenic or NSC-based chimeric mice expressing neurovirulent MLV Envs have yet to provide a clear resolution to this question [11-14].

We have previously demonstrated that over expression of the CasBrE Env protein, either SU or SU/TM, from engrafted NSCs does not induce acute spongiform pathology. Moreover, we have shown that dissemination of the CasBrE *env *gene from transplanted packaging/producer NSCs also does not cause spongiosis, despite Env expression within host microglia, a major CNS MLV target. Therefore, we were interested in examining whether additional retroviral proteins might also need to be delivered to host cells to induce neuropathogenic changes. To address this question, we have been exploring an NSC-based strategy wherein a "non-neurovirulent" amphotropic virus, 4070A, is used to pseudotype and *trans *complement the packagable CasBrE *env *vector, CasE, which encodes both the SU and TM subunits. The expectation being that if CasBrE *env *alone is not sufficient for disease, supplying Gag-pol elements in *trans *will provide the missing components. To address whether Env needed to be membrane associated, as has been reported for prions [15], we also investigated the effects of pseudotyping CasES, a vector that encodes CasBrE SU without the Env TM subunit. However, it is important to note that CasES expression from NSCs results in SU protein that is not able to efficiently bind to its cognate receptor, mCAT-1, a murine cationic amino acid transporter [16], when assessed by superinfection interference [11]. Why the CasBrE SU in the absence of the TM subunit had lost receptor binding activity despite the preservation of the RBD sequences in the N-terminal half of SU was not clear. Nonetheless, the loss of receptor binding might allow us to also investigate whether this activity could also be important for neuropathogenesis. However, before we could address these questions *in vivo*, we needed to examine whether 4070A Env and the CasBrE SU interacted within the engineered NSCs, as previous reports have demonstrated that the Env RBD domain could bind and *trans *complement Env proteins whose RBDs had been deleted or replaced with an alternative receptor specificity [17]. This possibility becomes increasingly important when considering how viral elements engineered into transplanted NSCs and delivered to the CNS could interact to facilitate acute spongiform neuropathogenesis. In this report, we show that CasBrE SU and 4070A proteins physically interact upon synthesis. This interaction results in CasBrE SU expression on the cell surface and facilitates its incorporation into viral particles. In addition, the interaction results in the restoration of CasBrE SU-mCAT-1 binding activity capable of establishing superinfection interference and facilitating ecotropic receptor-dependent virus infection. The finding that SU folding can be affected by protein-protein interactions has significant implications for understanding how NSC-mediated viral pseudotyping and *trans *complementation in the brain act to precipitate spongiform neurodegeneration.

## Results

### CasBrE SU is expressed on the cell surface upon NSC infection with an amphotropic helper virus

**Figure 1:**
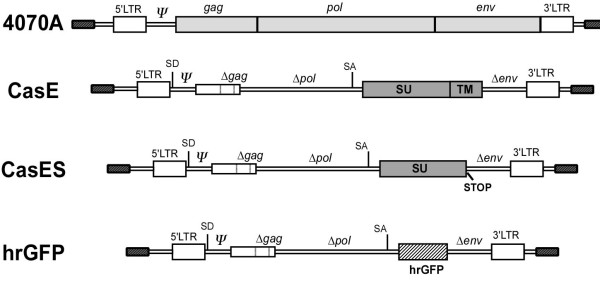
**Structures of the amphotropic virus and retroviral vectors introduced into NSCs for pseudotyping and *trans *complementation analysis of CasBrE *env *genes**. C17.2 NSCs were transduced with the pSFF-based retroviral vectors encoding CasBrE SU/TM (CasE), CasBrE SU (CasES), or the humanized *renilla *green fluorescent protein (hrGFP; striped) followed by infection with the amphotropic virus 4070A. The complementing viral structural elements are shown as filled regions for the 4070A virus and CasE and CasES vectors. The hrGFP vector served as a vector control that could be followed by fluorescence microscopy. Vector elements designated with a *Δ *represent deleted retroviral structural elements. *Ψ *indicates the presence of retroviral packaging sequences. SD, Splice donor. SA, Splice acceptor. LTR, Long terminal repeat.

**Figure 2:**
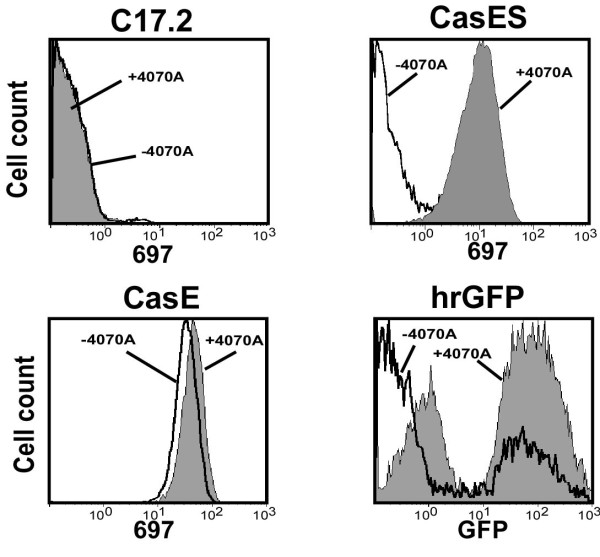
**FACS analysis of control, CasE, CasES, and hrGFP-vector transduced C17.2 NSCs, with and without 4070A infection**. The FACS analysis of CasES- and CasE-NSCs shown was limited to unpermeabilized cells in order to show cell surface CasBrE Env expression. CasBrE Env expression levels were detected via staining with the monoclonal antibody designated 697 [32]. GFP expression was detected directly.

To assess the feasibility of using a "non-neurovirulent" amphotropic helper virus, to disseminate and *trans *complement CasBrE *env *vectors within the brain by transplanted NSCs, C17.2 NSCs [18] were transduced with retroviral vectors encoding CasBrE SU (pCasES), CasBrE SU/TM (pCasE), or humanized *Renilla *green fluorescent protein (phrGFP), and subsequently infected with the 4070A retrovirus (Figure 1). The resulting cells were initially characterized by fluorescence activated cell sorting (FACS) for cell surface CasBrE Env expression (Figure 2). Importantly, no CasBrE Env immunostaining was observed in control NSCs with or without 4070A infection; however, CasE-NSCs, showed significant CasBrE Env expression both in the presence and absence of 4070A infection, with a small but reproducible increase in cell surface CasBrE Env after 4070A infection. Surprisingly, 4070A infection of CasES-NSCs resulted in the appearance of CasBrE Env on the cell surface, rather than being simply secreted into the medium. This result suggested that 4070A proteins interact with and *trans *complement CasBrE SU in a way that restores the native cellular distribution observed when the protein is made as the SU/TM polyprotein.

For vector pseudotyping controls, C17.2 NSCs were transduced with vectors encoding GFP. However, due to a lack of stable GFP expression, they could not be directly compared with CasBrE Env encoding vectors and their utility *in vivo *would be similarly limited. In this regard, GFP-sorted NSCs transduced with hrGFP (from *Victoria*; Stratagene) showed that within 10 days after isolation less than 50% of this population exhibited persistent GFP expression (Figure 2, hrGFP, -4070A). The loss of GFP expressing cells reoccurred even after repeated (3×) FACS selection. Surprisingly, infection of these cells with 4070A resulted in a higher level of cells persistently expressing GFP (+4070A), which was maintained through multiple cell passages without reselection. In parallel experiments with NSCs transduced with *Aequorea *EGFP, we were unable to generate NSCs that stably expressed GFP, regardless of whether they were infected with 4070A.

### 4070A infection of CasBrE Env expressing NSCs influences Env and Gag expression levels

**Figure 3:**
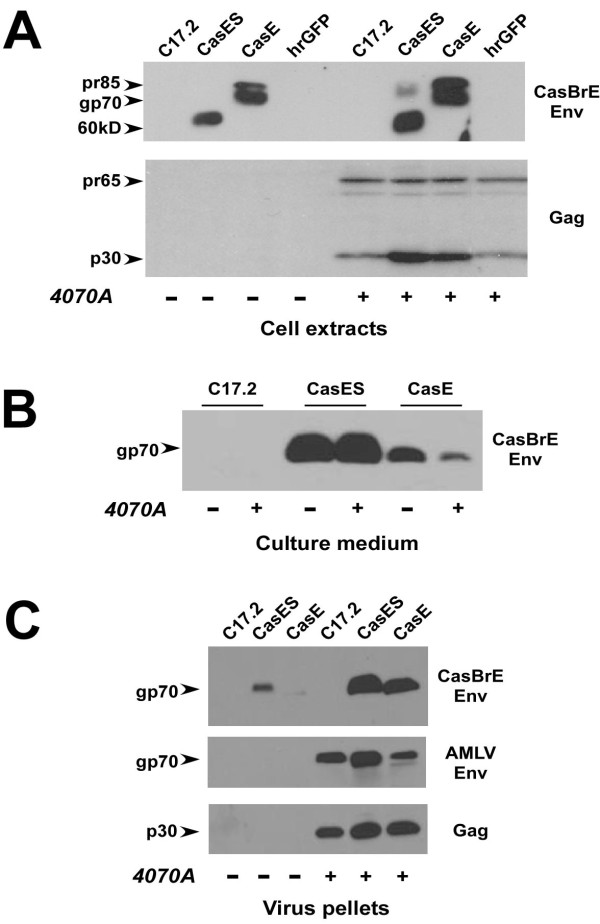
**Western blotting analysis of vector transduced NSCs without (-) and with (+) 4070A infection**. **A**. Equivalent whole cell extracts of control C17.2 NSCs and those expressing CasE, CasES or hrGFP, with and without 4070A infection, were assessed for CasBrE Env expression (αCasBrE Env; top) and Gag (αGag; bottom) after separation on 9% SDS-PAGE gels. CasES-NSCs produce a 60 kD Env isoform in contrast to the gp70 and precursor (pr85) isoforms made in CasE-NSCs [11]. The 60 kD isoform likely represents minimally glycosylated Env protein [40]. Note that CasBrE Env levels were elevated with 4070A infection, and p30gag levels are elevated in 4070A infected NSCs possessing CasBrE Env vectors. **B**. Examination of CasBrE Env released into the culture medium ± 4070A infection. Note that CasBrE gp70 release is reduced in the presence of 4070A infection for CasE-NSCs but not CasES NSCs. **C**. NSC culture supernatants were subjected to ultracentrifugation and virion pellets were assessed for CasBrE Env (top), 4070A Env (middle) and Gag (bottom). All blots were run in triplicate using equivalent protein loads that were confirmed by coomassie blue staining of equivalent gels run in parallel. Representative examples are shown.

To characterize the potential influence of 4070A virus infection on CasBrE Env expression in NSCs, equivalent cell extracts, culture supernatants, and pelleted culture supernatants were analyzed by Western blotting for CasBrE Env and 4070A proteins (Figure 3). This analysis showed that 4070A infection resulted in a reproducible increase in the levels of cell-associated CasBrE Env protein (Figure 3A). Specifically, NSCs expressing CasE showed an increase in precursor (pr85) and processed (gp70) Env isoforms, while CasES expressing NSCs showed a similar increase in the 60kD Env isoform that is associated with these cells {cf. [11]}. In addition, CasES NSCs infected with 4070A expressed a small amount of higher apparent molecular weight Env proteins resolving at 80-85 kD. Analysis of CasBrE Env protein released into the medium from the engineered/infected NSCs (Figure 3B) showed that those cells expressing CasES released significant levels of gp70, which was not appreciably affected by 4070A infection. In contrast, 4070A-infected CasE-NSCs showed a reproducible reduction in CasBrE gp70 release into the medium post infection. It was not specifically explored whether this decrease resulted from the increase in cell-associated CasBrE Env protein noted above. Parallel analysis of Gag expression in the engineered NSCs with and without 4070A infection (Figure 3A, lower panel) showed p30 Gag expression in all 4070A-infected NSC cultures. Notably, after infection with 4070A, p30Gag appeared to be expressed at higher levels in CasE- and CasES-NSCs. Increased Gag levels appeared to positively correlate with CasBrE Env protein, rather than simply the pSFF vector, as hrGFP-NSCs infected with 4070A showed levels of Gag protein indistinguishable from 4070A-infected control NSCs.

To assess whether both CasBrE and 4070A Env proteins were incorporated into viral particles, culture supernatants from the control, CasES- and CasE-NSCs with and without 4070A, were subjected to centrifugation to pellet virus. The sedimented fractions were assessed for CasBrE and 4070A Env by immunoblot. Figure 3C, **far right panels **show that both 4070A and CasBrE gp70 s were associated with p30Gag in the pellets indicating that both 4070A and CasBrE Envs were being incorporated into virions. This is especially noteworthy for the CasES+4070A-NSCs as CasBrE SU has no inherent membrane interacting domain and thus was likely interacting with 4070A proteins to achieve virion incorporation. The small amount of CasBrE SU noted after sedimentation of the CasES supernatant without 4070A represents trace amounts of medium containing high levels of SU rather than the sedimentation of SU aggregates.

### CasBrE SU physically interacts with 4070A on the plasma membrane of NSCs

**Figure 4:**
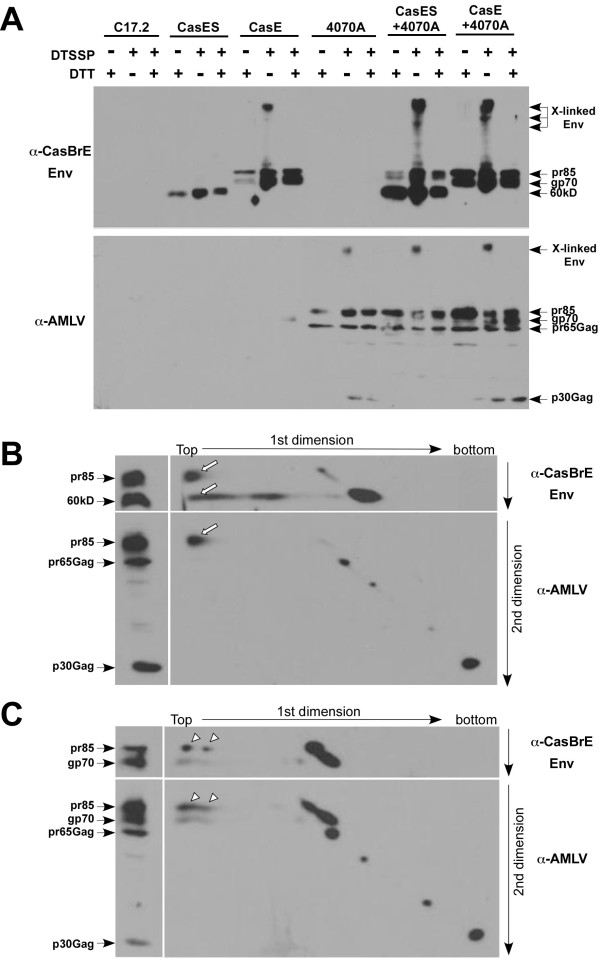
**Cell surface cross-linking reveals direct CasBrE Env and 4070A interactions in pseudotyping NSCs**. **A**. Treatment of control, 4070A virus, or vector transduced C17.2 NSCs with the water soluble chemical cross-linker DTSSP to assess cell surface interactions between CasBrE SU or SU/TM and 4070A Env. Extracts from cross-linked cells were treated with or without dithiotheitol (DTT), separated by SDS-PAGE on 8% gels, followed by immunoblotting first for CasBrE Env (A-C, top panels; 697) followed by stripping and reprobing for 4070A viral proteins (A-C, lower panels; pig anti-AmLV). Cross-linked complexes (X-linked Env) appear at the top of the blots as slow mobility bands or smears, which were converted by DTT treatment to molecular mobility patterns consistent with un-crosslinked samples. **B**. Sequential immunoblotting of cross-linked samples from CasES+4070A-NSCs separated by non-reducing SDS-PAGE (without DTT) in the first dimension (horizontal) followed by reducing SDS-PAGE (with DTT) in the second dimension (vertical). The same sample was also treated with DTT and only run in the second dimension indicated by the separate lane (left columns) to mark the migration of the different immunoreactive components. **C**. Non-reducing/reducing two-dimensional SDS-PAGE analysis of crosslinked CasE+4070A NSC extracts. Arrows and arrowheads indicate the presence of the Env species that co-migrated in the high molecular weight cross-linked complexes.

The appearance of CasBrE SU on the cell surface and in virions after NSC infection with 4070A suggested that the 4070A Env interacts with CasBrE SU to tether the latter protein to the plasma membrane. To provide support for this idea, NSCs were exposed to DTSSP, a water soluble, thiol-cleavable crosslinker, to stabilize cell surface protein-protein interactions. Cellular extracts were then subject to Western blotting using both CasBrE Env and amphotropic virus specific antibodies to investigate whether Env hetero-oligomers were formed. As shown in Figure 4A, low mobility (high apparent molecular weight) CasBrE Env and amphotropic virus-specific bands were detected in CasE-, 4070A-, CasES+4070A-, and CasE+4070A-NSC cultures in the presence of the DTSSP, which were eliminated by reduction with dithiothreitol (DTT). Importantly, no crosslinked CasBrE SU complexes were detected in DTSSP-treated CasES-NSC extracts, consistent with the idea that CasBrE SU multimers or mixed protein complexes were absent from the cell membrane. Because the cross-linked CasES+4070A low mobility bands observed with CasBrE-specific and AMLV-specific antibodies were observed to localize to the same spots when the blots were overlayed, the results suggested that CasBrE SU and 4070A Env were inter-cross-linked on the cell membrane. Similar findings were observed for low mobility bands for CasE+4070A-NSCs, however, the potential for interaction here are less clear here since complexes with low mobility can be seen with CasE- and 4070A-NSCs alone.

To further examine what proteins were contained in the low mobility (high molecular weight), DTSSP-crosslinked bands from CasES+4070A- and CasE+4070A-NSCs, extracts were subjected to non-reducing SDS-PAGE in the first dimension followed by reducing SDS-PAGE in the second dimension, with subsequent analysis by Western blotting for CasBrE Env and 4070A viral proteins. Two CasBrE immunoreactive bands corresponding to pr85 and 60 kD proteins, and one amphotropic pr85 Env band (Figure 4B, **arrows**) were readily detected in the low mobility cross-linked bands obtained from CasES+4070A cells; again, suggesting that CasBrE SU and 4070A Env proteins may directly interact. Similar results were observed for CasE+4070A NSCs with both pr85 and gp70 bands being found in the low mobility bands (Figure 4C), however, because CasE-NSCs and 4070A-NSCs alone also show low mobility complexes after DTSSP treatment, it was not possible to distinguish between homo- versus hetero-oligomers upon dissociation in the presence of reducing agent.

### 4070A helper virus-infected NSCs efficiently transduce viral and vector genomes

**Figure 5:**
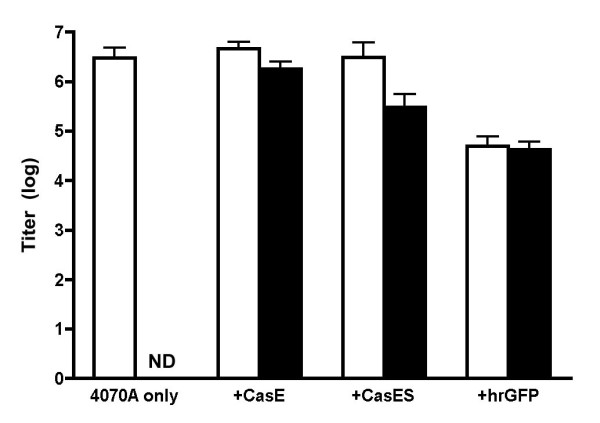
**Viral pseudotyping from 4070A-infected NSCs**. Virus titration assays (VTAs) were performed on *dunni *fibroblasts using culture supernatants derived from 4070A infected CasE-, CasES-, hrGFP- and control-C17.2 NSCs for total virus (white bars) and vector only encoding virus (black bars). ND = not detected. Total virus was detected with an Env specific antibody, 83A25 that recognizes both 4070A and CasBrE envelope proteins. CasBrE Env encoding viruses were detected by monoclonal antibody 697 [32]. HrGFP encoding virus was determined by direct fluorescence detection of infected cells.

We next examined whether the NSC-generated virions were capable of transducing viral and vector genes to naïve targets. As shown in Figure 5, 4070A-infected NSCs generated total virus titers (**white bars**) greater than 10^6 ^focus forming units per ml (FFU/ml) for all control or vector engineered NSCs, except those containing hrGFP. The presence of CasES or CasE in the NSCs did not appear to alter infectious virus production from these cells, in seeming contrast to the presence of hrGFP. Infected NSCs examined for the production of virus encoding CasBrE-SU/TM, -SU, or hrGFP showed that all three vectors were incorporated into infectious virus (Figure 5, **black bars**). While the data suggest that the CasE vector was most efficiently pseudotyped by 4070A, the differences between groups were not statistically significant when analyzed by one way ANOVA at the 95% confidence interval.

### CasBrE SU-4070A Env interactions facilitate virus entry via the ecotropic receptor

**Figure 6:**
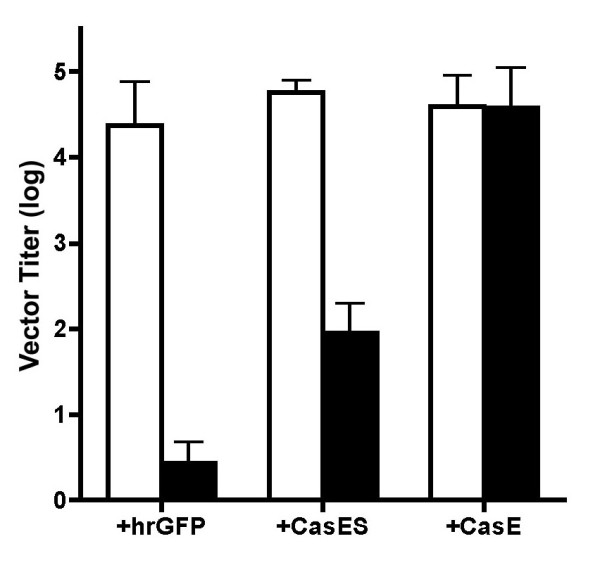
**CasBrE SU mediates virus entry via the ecotropic receptor**. Viral supernatants taken from 4070A-infected hrGFP-, CasES-, and CasE-NSCs were titered on NIH 3T3 fibroblasts cells with (black bars) and without (white bars) 4070A infection. The 4070A infection acts as a means to interfere with virus entry via the amphotropic receptor, PiT-2. CasBrE Env+ foci were detected using monoclonal antibody 697 and hrGFP foci were detected by direct viewing of target cell fluorescence. Assays were run in triplicate and error bars represent the standard deviation of the log of the vector titers.

To assess whether virion-associated CasBrE SU was capable of initiating virus entry via interactions with the ecotropic receptor, mCAT-1, rather than via the amphotropic receptor, PiT-2, supernatants from hrGFP+4070A-, CasES+4070A- and CasE+4070A-NSCs were examined in a virus titration assay using target fibroblasts (3T3 cells) that had been previously infected with 4070A (3T3Am) to establish amphotropic virus superinfection interference. Foci were scored based on the expression of the vector encoded gene products, CasBrE Env or hrGFP. As shown in Figure 6, supernatants from all three NSC lines exhibited vector titers of approximately 5 logs on normal 3T3 targets (white bars). The vector titer was not detectably diminished when supernatants from CasE+4070A-NSCs were tittered on 3T3Am targets, indicating that CasBrE SU/TM facilitates vector transmission even when the amphotropic receptor was blocked. In contrast, titers from CasES+4070A- and hrGFP+4070A-NSC supernatants were reduced by at least 3 orders of magnitude when assayed on 3T3Am cells (or dunniAm cells; not shown). Because hrGFP+4070A-NSC-derived virions do not possess an ecotropic Env protein to overcome amphotropic viral interference the reduced viral titers were expected, however, with CasES+4070A NSCs, the results suggest that 4070A Env-CasBrE SU interactions responsible for particle incorporation were inadequate to facilitate efficient free virus entry through mCAT-1.

**Figure 7:**
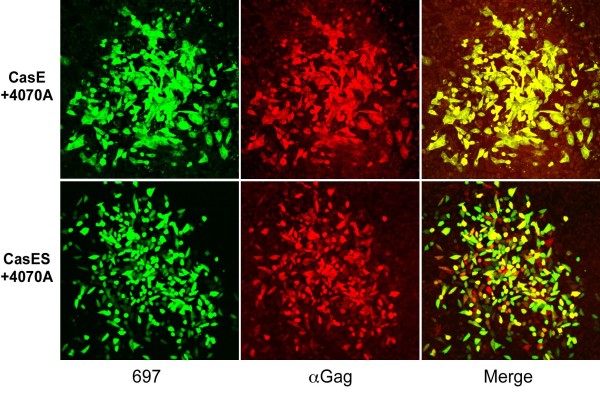
**Target cells are dually infected by 4070A and pseudotyped viral vectors**. Infectious centers (ICs) were generated by seeding 4070A-infected CasE- and CasES-NSCs with target dunni fibroblasts followed by double immunostaining for CasBrE Env (697; red) to indicate vector infection and Gag (αGag, green) to depict 4070A virus infection. Abundant colocalization in the target fibroblasts is shown in the merged images (Merge) indicating that both the viral vector and 4070A genes were efficiently delivered to target cells. Bar = 100 μm.

Because retroviral SU-TM interactions are generally considered to be meta-stable in the absence of a disulfide cross-link, and in the case of CasBrE SU-4070A SU/TM, hetero-oligomeric interactions may be even less stable, we asked whether 4070A Env-CasBrE SU interactions might facilitate more efficient retroviral vector transfer via CasBrE Env-mCAT-1 interactions under conditions of direct cell-cell contact. Therefore, the 4070A-infected viral vector expressing NSCs were examined by infectious center assay on target fibroblasts with and without amphotropic virus infection. As shown in Table 1, the CasE+4070A, CasES+4070A-, and hrGFP+4070A-NSCs scored very efficiently as infectious centers on naïve dunni fibroblasts (cf., Figure 7), and suggested that essentially all the helper virus-infected NSCs appeared capable of efficiently delivering retroviral vectors to susceptible target cells *in vitro*, despite the variability in plating efficiency. Importantly, when these same NSCs were seeded onto 4070A-infected dunni fibroblasts (dunniAm), only hrGFP+4070A-NSCs failed to generate viral vector positive foci. CasES+4070A-NSC ICs were readily observed in dunniAm cultures, although the numbers were reduced from their value in naïve dunni cell cultures. In contrast, CasE+4070A-NSCs showed no decrease in IC titers on dunniAm cells. These results indicate that in the presence of the 4070A Env, CasBrE SU facilitates ecotropic entry, but not with the same efficiency as native CasBrE SU/TM.

**Table 1:** CasBrE Env-NSC mediated superinfection of amphotropic-restricted targets through cell-cell contact

**NSCs**	**Infectious Centers^a^**		**Plating Efficiency^b^**	**Infectious Center Efficiency^c^**	
			
	***Dunni***	***DunniAm***		***Dunni***	***DunniAm***
					
**hrGFP+4070A**	10.5 ± 1.3	< 0.05*	2.5 ± 1	420%	< 2%
**CasES+4070A**	67 ± 8.3	18 ± 3	16.5 ± 3	406%	109%
**CasE+4070A**	49 ± 5.0	64.7 ± 7.2	16.2 ± 3.8	302%	400%

a- Mitomycin C-treated 4070A-NSCs expressing GFP, CasE or CasES were counted and serial dilutions were seeded with 10^5 ^*dunni *fibroblasts. The number of foci expressing the viral vector once the Dunni cells reached confluence was evaluated and expressed as foci/per 100 NSCs seeded.

b- To determine the % plating efficiency, the mitomycin C-treated NSCs were counted and seeded as in "a" in the absence of *dunni *cells and the percentage of vector expressing cells present on these plates, versus the number seeded, was assessed at 48 hours.

c- IC efficiency is simply the number of foci/100 NSCs seeded divided by the plating efficiency expressed as a percentage.

*-The detection of an infectious center is limited by the maximum number of NSCs seeded per plate of targets. For these experiments it was limited to ≤ 2000 NSCs/plate.

### Helper virus-infected NSCs deliver both virus and vector to naive target cells

To assess whether the 4070A-infected vector-expressing NSCs could deliver both virus and vector to individual *dunni *fibroblasts to facilitate *trans *complementation within those targets, infectious center foci were assessed for CasBrE Env and 4070 Gag (Figure 7). Examination of the merged images showed that virtually all the Env+ cells (green) were also Gag+ (red) with CasE+4070A-NSCs. In foci generated from CasES+4070A NSCs approximately 60% of cells were double positive, whereas, approximately 15% were singly positive for CasBrE SU, and 25% singly positive for Gag. Analysis of hrGFP+4070A NSCs gave results similar to those generated by the CasES vector with between 50-70% of cells in foci expressing GFP and Gag (not shown).

### 4070A helper virus infection of CasBrE Env expressing NSCs does not result in the generation of recombinant replication competent CasBrE virus

**Figure 8:**
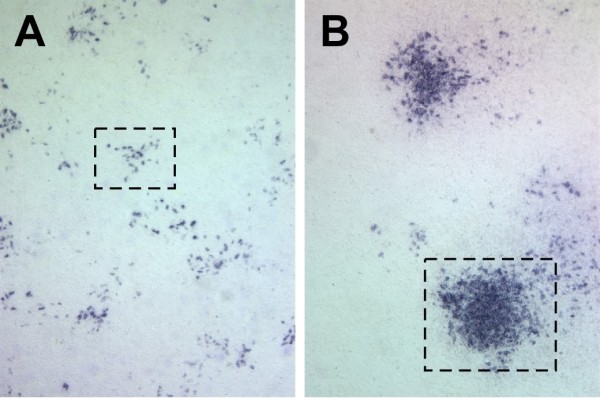
**Foci morphology of infectious virus derived from pseudotyping NSCs**. **A.** Representative foci (boxed areas) from cells infected with supernatants from CasE+4070A-NSCs stained for CasBrE Env showing non-contiguous foci morphologies indicative of defective replication incompetent virus. No contiguous 697 foci were noted under conditions where single-hit infection predominated, indicating that replication competent recombinant CasBrE viruses did not readily arise from CasE+4070A-NSCs or CasES+4070A-NSCs. **B**. Representative foci from fibroblasts infected with CasE+4070A supernatants stained with monoclonal antibody 83A25 which recognizes both 4070A and CasBrE Envs. This image illustrates both non-contiguous and contiguous foci morphologies, indicative of defective and replication competent viruses, respectively.

Despite findings indicating that MLVs infrequently co-package retroviral genomes arising from different DNA transcriptional templates [19], the potential still existed for possible recombination between the helper virus and the retroviral vectors containing CasBrE Env that could be amplified. Thus, NSC supernatants were evaluated for the presence of replication competent CasBrE Env-encoding virus by examining foci contiguity at virus dilutions where single-hit kinetics predominate as previously outlined [14]. As shown by the example in Figure 8, **left panel**, all CasBrE Env positive (697+) foci appeared non-contiguous (small box), indicating that the initial virus infectious event did not spread cell-to-cell from the primary target, but rather expanded due to target cell division and migration. The lack of detectable contiguous foci suggests that if recombination did occur it did so at a very low frequency, given that more than 1000 foci were examined. Similar results were observed for supernatants from infected NSCs expressing CasES (not shown). In contrast, examination of parallel assays at the same time, with antibody reactive to both 4070A and CasBrE Envs (83A25), showed abundant contiguous foci (**box area, right panel**) indicative of cell-to-cell spread of the replication competent 4070A virus.

### CasBrE SU-4070A Env interactions facilitate efficient binding and blocking of mCAT-1 in NSCs

**Figure 9:**
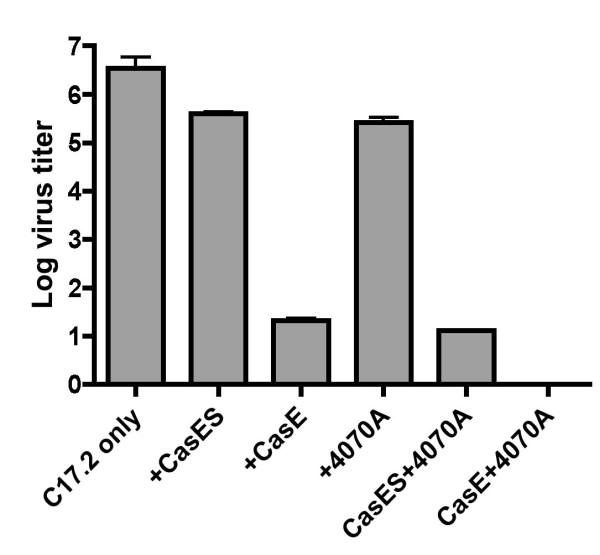
**Assessment of CasBrE Env binding to mCAT-1 by superinfection interference**. Control, CasES- and CasE-C17.2 NSCs with and without 4070A infection were subjected to infection by the ecotropic virus, Fr57E, to assess the ability of NSCs-expressing CasBrE Env or SU to block Fr57E entry through the ecotropic virus receptor mCAT-1. Viral foci on NSCs were detected with monoclonal antibodies 48, 500, and 720 that are specific for the Friend virus Env [38,39]. Error bars represent the standard deviation of the log of the Fr57E virus titers. N = 4 for all the samples.

The interactions between CasBrE SU and 4070A Env appear to at least partially restore CasBrE SU ecotropic receptor binding and fusion activity, so we further explored the extent to which this interaction could influence superinfection interference by binding to mCAT-1 within the NSCs and prevent infection by a second ecotropic virus, Fr57E. The results, shown in Figure 9, indicate that CasES expression alone within NSCs only reduced superinfection by the ecotropic virus, Fr57E, by ~1 log, whereas, CasE expression reduced infectivity by ~5 logs compared to control NSCs. 4070A infection of NSCs also reduced the Fr57E titer by ~1 log, however, 4070A infection of CasES- and CasE-NSCs reduced Fr57E titers by 5 and 6 logs respectively. The 4 log increase in ecotropic retrovirus superinfection interference when 4070A and CasBrE SU are co-expressed suggests that 4070A Env-CasBrE SU interactions result in restoration of near native CasBrE SU-mCAT-1 binding interactions analogous to CasBrE SU/TM. Whether such intracellular Env-receptor interactions play a role in retroviral neuropathogenesis has not been specifically investigated, but in the helper virus paradigm being explored herein, CasBrE SU appeared capable of carrying out the same functions as SU/TM, albeit with more limited specific activity, and only in the presence of the *trans *complementing protein from 4070A.

## Discussion

In this report we examined the capacity of the 4070A amphotropic virus to *trans *complement and pseudotype CasBrE *env *vectors when co-expressed in NSCs as a prelude to transplanting these cells in the CNS in order to define what retroviral elements and interactions were required to induce spongiform neurodegeneration. This analysis revealed that the engineered NSCs could indeed deliver both the *env *vector and complementing 4070A virus to target cells in culture, in the absence of detectable recombinant virus. Given these results, the potential exists for exploiting this capacity for NSC-mediated pseudotyping and *trans *complementation to reveal insights into the elements and activities involved in retroviral neurovirulence and their capacity to target certain CNS cell types. However, our *in vitro *analysis herein, also revealed how viral protein-protein interactions might be expected to alter the interpretation of the *in vivo *experiments where these tools are employed. Specifically, we showed that direct heteromeric interactions between 4070A Env and the protein product of the CasES vector, CasBrE SU, restored CasBrE SU's capacity to associate with the plasma membrane, become incorporated into viral particles, bind to the ecotropic receptor and trigger virus fusion.

We previously reported that when CasBrE SU is made in the absence of TM subunit sequences, the protein possessed little ecotropic receptor binding activity and was secreted from the cell [11]. Presumably the absence of TM sequences in the Env precursor protein prevented the normal folding of the CasBrE receptor binding domain (RBD) found within SU. The loss of receptor binding activity for CasBrE SU was not predicted given the findings of Davey et al. showing that eukaryotic cell synthesis of the N-terminal one third of the Friend virus SU results in receptor binding activity equivalent to the full length protein (SU/TM) [20]. Spatial modeling of the CasBrE RBD sequence, based on the crystal structure defined for the RBD from the Friend virus [21], predicts a similar structure for CasBrE RBD (unpublished observations). Thus, sequences in the latter two thirds of SU may negatively influence the proper folding of the CasBrE SU in the absence of TM sequences. It is clear from studies by Lavillette et al., that there are critical interactions between the C-terminal domain of SU and the RBD that affect various Env entry activities [22]. Thus, the results here suggest that, at least for CasBrE Env, sequences within TM may help to facilitate the proper folding of these domains in CasBrE SU.

Given the virtual absence of CasBrE SU receptor binding activity and cell surface expression originally observed in CasES NSCs, we predicted that 4070A infection of CasBrE SU-expressing NSCs would result in the production of virus that would demonstrate only amphotropic tropism while packaging either 4070A or CasES genomes. It was therefore surprising to observe CasBrE SU on the cell surface of CasES+4070A-NSCs in the absence of any recombinant virus. The follow-up cross-linking, sedimentation, infection and superinfection analyses demonstrated that direct 4070A Env-CasBrE SU interactions increased ecotropic receptor interactions by 10,000 fold and facilitated SU incorporation into virions that could activate fusion activity.

How the lost SU activities were restored is not clear, but it is of interest to speculate that the 4070A Env may have acted as a folding template for CasBrE SU during its synthesis through the secretory pathway. In this way, Env would be analogous to the prion protein where the scrapie form is able to direct the folding and ultimate conformation of the unfolded protein through direct interactions. This idea appears to be a reoccurring theme in a diverse array of infectious, sporadic and genetic neurodegenerative diseases often characterized by protein aggregation. While retroviral neuropathogenesis is not characterized by identifiable protein aggregates, their ability to form multimers may provide a structural link between such diseases. The possibility that template-based protein folding extends to retrovirus-induced neurodegeneration such as HTLV-1 associated myelopathy or HIV associated dementia is provocative.

An alternative to the template-based folding idea is that SU interactions with 4070A SU or TM may have facilitated more efficient or prolonged SU interactions with ER chaperones through non-covalent tethering of SU to the membranes in the secretory pathway. However, we observed that CasBrE SU synthesized alone appears to be fully glycosylated and of the correct molecular size upon its secretion into the medium, suggesting that it has been routed and processed through the requisite ER-golgi protein quality control machinery that includes lectins and sugar processing enzymes (reviewed in [23-25]). Whether the addition of a simple transmembrane domain or a glycophosphatidyl inositol linker to CasBrE SU would result in a similar restoration of CasBrE SU binding to mCAT-1 might help distinguish between these two possibilities.

The observation that correctly folded CasBrE SU could facilitate ecotropic receptor binding and entry in the presence of amphotropic Env is consistent with the modular nature of type-C retroviral Envs described by Barnett et al. [17]. These investigators showed that the addition of soluble Friend RBD could restore infectivity when the RBD was deleted from ecotropic, amphotropic, or xenotropic Envs. Morever, this group showed that RBD could enhance infectivity 50-fold when Env RBD sequences were replaced by erythropoietin as an alternate receptor specificity. Herein, CasBrE-SU facilitated ecotropic receptor-mediated virus fusion indicating that the CasBrE SU induced conformational changes in 4070A derived-TM trimers required for membrane fusion. Whether this involved CasBrE SU binding to functional 4070A SU/TM monomers, dimers, or trimers, or represented CasBrE SU displacement of 4070A SU to interact with TM is not clear. The cross-linking studies appear to suggest that heteromeric CasBrE-4070A SU-SU interactions were occurring, but the exact nature of the fusigenic structures is a matter of conjecture at this stage. Interestingly, a previous report by Yang et al. suggests that ecotropic and amphotropic Env hetero-trimers are not formed when co-expressed in the same cell, however, this apparent discrepancy could be accounted for by the fact that differing ecotropic viruses were employed in the two studies. In this regard, the CasBrE virus was initially co-isolated from the wild along with amphotropic viruses [26], suggesting that the two viruses likely co-evolved. Nonetheless, since we did not observe full restoration of CasBrE Env binding and fusion activity in this complementation paradigm, it suggests that CasBrE SU-4070A TM heterodimer formation was either not highly efficient or that misfolded CasBrE SU might also compete with natively folded SU for association with 4070A SU/TM or TM proteins. It is clear that a more detailed analysis of the protein complexes being formed will be required to reveal insight into the nature of the specific protein-protein interactions taking place.

Given that previous experiments investigating prion protein neuropathogenesis have demonstrated that the glycophosphatidyl inositol membrane anchor was required for prion protein induced neurodegeneration but not replication [15], we initially thought that NSC-mediated 4070A delivery of CasES to CNS cells would allow us to assess whether ecotropic receptor binding activity and/or Env membrane association were required for MLV-induced spongiform neurodegeneration. However, since these Env activities were restored by CasBrE SU-4070A Env interactions, these questions cannot be effectively addressed using this helper virus pseudotyping approach. Nonetheless, the capacity for *trans *complementation *in vivo *examined in the companion study was able to show that ecotropic entry is a critical step in the pathogenic process, and that the "non-neurovirulent" virus amphotropic virus contains neurovirulent elements that could be revealed by such an analysis. Perhaps more dramatically, these findings of Env-SU interactions highlight the difficulty in assessing which *in vivo *Env interactions are responsible for the induction of neuropathogenic changes given that the mouse genome, like that of other mammals, is replete with retroviral sequences. Ruling out that exogenous viral proteins interact with host retroviral elements to cause disease may be a Sisyphean task.

## Conclusions

The findings presented here show that co-expression of 4070A and CasBrE SU results in a physical interaction between 4070A Env and CasBrE SU. This interaction facilitates CasBrE SU retention on the plasma membrane, incorporation into viral particles and restoration of its ability to interact with its receptor, mCAT-1. Moreover, Env-SU oligomers were capable of initiating ecotropic receptor-dependent TM-mediated fusion events, despite reduced efficiencies in free virus. Thus, the results indicate that heterotropic Env-SU interactions that occur during protein synthesis facilitate CasBrE SU folding events and interactions with TM such that ecotropic viral binding and membrane fusion events were almost fully restored. This template based folding process is reminiscent of prion formation, where one protein conformation acts as a scaffold for the folding of a second protein of similar primary structure. These findings support the idea that protein scaffold-based protein folding represents an inherent feature of neuropathogenic proteins that appears to extend to retroviral Envs.

## Methods

### DNA Constructs

The CasBrE env expression vectors CasE (SU/TM), CasES (SU), described previously [11], contain the *env *gene derived from the CasBrE molecular clone 15.1 [27,28] (GenBank accession number X57540) inserted into the pSFF vector [29]. In order to assess effects associated with vector alone, a pSFF-based vector encoding humanized GFP from Renilla (hrGFP, Stratagene) was generated by introducing a Xho I and Sal I fragment from pFB-hrGFP (Promega) into the multiple cloning site of pSFF.

### Cells

C17.2 neural stem cell lines with and without expression of CasBrE SU or SU/TM have been described previously [11,30] and were grown on Primaria dishes (Falcon) in DMEM supplemented with 10% fetal bovine serum and 100 U of penicillin and streptomycin per ml. *Mus dunni*, NIH 3T3 fibroblasts, and PT67 packaging/producer cells (Invitrogen) were maintained in DMEM with 10% new born calf serum and 100 U of penicillin and streptomycin per ml on Nunclon D™ dishes (Nunc).

HrGFP-NSCs were generated by exposing C17.2 cells to hrGFP virus at a multiplicity of infection of 1 in the presence of 8 μg/mL polybrene. Positive NSCs were sorted by flow cytometry with the brightest 10% selected and used for subsequent analysis. C17.2 NSCs with and without SU, SU/TM, hrGFP or 15-1EIH were infected with the 4070A virus at an MOI of 1 in the presence of 8 μg/mL polybrene, and passaged 4 times prior to analysis for CasBrE Env and hrGFP expression and 4070A virus pseudotyping.

### Viruses

4070A amphotropic virus stocks were generated by transfecting the permuted proviral clone p4070A [31] into NIH3T3 cells. Virus encoding hrGFP was generated by co-transfection of PT67 packaging producer cells (Invitrogen) with pCDNA3.1-hygro and phrGFP followed by collection of viral supernatants from hygromycin selected cultures. Similarly, CasE, CasES, and SFF-FE virus stocks were generated by transfection of PT67 cells with the corresponding plasmid.

### FACS analysis

NSCs were removed from tissue culture plates by using 0.05% trypsin with EDTA (Gibco), quenched with DMEM+10% NCS, washed with PBS, and incubated at 4°C for 30 minutes with the mouse monoclonal antibody 697 for detecting CasBrE Env [32], or mouse monoclonal 48 for Friend 57 Env. Cells were washed three times with PBS, and incubated with Alexa Fluor 488-conjugated goat anti-mouse IgG (Molecular Probes) for another 30 min before analysis. HrGFP expression within NSCs was analyzed directly. Analysis and sorting was carried out on an Altra Flow Cytometer (Beckman/Coulter).

### Western blotting

Tissue culture cell extracts were generated as previously outlined [11,33] and equivalent samples were separated by 8-10% SDS-PAGE, transferred to nitrocellulose membrane, blocked with 5% nonfat milk in TBS. To detect CasBrE Env and SU mouse monoclonal antibody 697 was used. Gag proteins were detected using rat anti-capsid R187 monoclonal antibody or rabbit anti-capsid antisera R3. Amphotropic viral proteins were detected using swine anti-AMLV serum (NCI). Primary antibodies or antisera was detected using horseradish peroxidase (HRP)-labeled goat anti-mouse IgG (Southern Biotechnologies), HRP-rabbit anti-swine IgG (RDI), HRP-donkey anti-rat IgG (Jackson Immunoresearch), or HRP-goat anti-rabbit IgG (Southern Biotechnologies), which were detected by chemiluminescence using ECL reagents (Pierce). Equivalent amounts of proteins were loaded for each tissue or cell sample and which was confirmed by Coomassie blue staining of a gel run in parallel in each experiment and by post transfer staining of the gel utilized in the transfer.

### Chemical cross-linking of cell surface proteins

The water soluble chemical cross-linker dithiobis sulfosuccinimidylpropionate, DTSSP (Pierce), was dissolved in PBS (pH 7.2) to a concentration of 1 mM just before use. Full confluent cells were washed with PBS three times and then incubated with 1 mM DTSSP solution at room temperature for 30 min, followed by washing three times with Tris-buffered saline (pH 7.5). DTSSP treated cells were extracted by using lysis buffer containing 0.1% Triton X-100 as previously described [33]. Cleared crosslinked samples were analyzed for viral proteins by Western blotting after non-reducing sodium dodecylsulfate polyacrylamide gel electrophoresis (SDS-PAGE) in the first dimension and reducing SDS-PAGE in the second dimension. Briefly, equivalent samples were boiled in SDS-PAGE sample buffer without DTT and resolved on an 8% gel and analyzed by Western blotting using monoclonal 697 for CasBrE Env and swine anti-AMLV (NCI) for 4070A proteins. To assess the composition of the cross-linked viral proteins, first dimension gel strips were equilibrated with sample buffer containing 100 mM DTT at room temperature for 15 min, positioned on the top of a second 8% gel, and then subjected to electrophoresis in the orthogonal directions and again analyzed for viral proteins by sequential immunoblotting.

### Virus titration assay

Virus titers of tissue culture supernatants were determined by a focus forming assay on *dunni *cells [34]. Supernatants collected from confluent cell cultures were frozen at -80°C before analysis. Viral Env positive foci were detected by using CasBrE Env specific MAb 697 or rat MAb 83A25 [35] which recognizes an epitope common to all known endogenous and most exogenous MLV Envs. Alkaline phosphatase-labeled anti-mouse IgG or anti-rat IgG antibodies (Promega) and Western Blue stabilized substrate for alkaline phosphatase (Promega) were used. GFP positive foci were determined directly under a fluorescence microscope. Virus foci were scored based on their morphology as contiguous or non-contiguous, the latter being consistent with defective virus [14]. To determine CasBrE SU positive or Gag positive foci, cells were permeabilized with ice cold methanol for 5 min before staining.

### Infectious center assay

In order to assess NSCs' ability to disseminate viral vectors by cell-to-cell contact, infectious center assays were performed as outlined by Czub et al. [36] with minor modifications. Briefly, transduced C17.2 NSCs were treated with mitomycin C (Fisher) at 10 μg/ml in DMEM medium at 37°C for 2 hr, washed with PBS three times, trypsinized and resuspended in DMEM+10% FBS. Treated NSCs were seeded into TC-6 tissue culture plates using serial dilutions yielding from 100 to 2000 cells per well. Wells were also seeded with 1 × 10^5 ^*dunni *target cells per well. Once the *dunni *cells reach confluent, 697, R187 and MAbs 720/500/48 were used to detect CasBrE Env, amphotropic Gag and Friend Env expression respectively, followed by Alexa Fluor 488 goat anti-mouse IgG, Alex Fluor donkey 488 anti-goat IgG and Alexa Fluor 594 donkey anti-rat IgG (Molecular Probes) respectively, and foci were examined under the epifluorescence microscopy. Images were merged to colocalize CasBrE Env or SU and 4070A Gag expression in some instances. Plates without *dunni *cells were used to assess the plating efficiency of the mitomycin-treated NSCs and to normalize the number of foci generated.

To study whether CasES+4070A-NSCs were capable of delivering virus via the ecotropic receptor by cell-cell contact, a similar assay was performed in which naïve *dunni *and amphotropic 4070A virus infected *dunni *(DunniAm) cells were used as target cells. CasBrE SU positive foci were determined. As controls, CasE-4070A-NSCs and hrGFP-4070A-NSCs were analyzed in parallel.

### Superinfection interference assays

To study whether CasBrE SU and Env could facilitate virus entry via the ecotropic receptor in the presence of the amphotropic 4070A virus, the superinfection interference assay was carried out as described previously [37]. Briefly, NSCs were infected with serially diluted ecotropic MLV Fr57E in the presence of polybrene (8 μg/ml). Friend Env positive foci were evaluated by its' specific monoclonal antibodies 500, 48 and720 [38,39], followed by Alexa Fluor 488 goat anti-mouse IgG (Molecular Probes). GFP positive foci were counted under a fluorescent microscopy directly.

To investigate whether the viruses generated by CasES+4070A-NSCs was capable of infecting targets via the ecotropic receptor, a similar method was carried out, in which 4070A virus infected-NIH 3T3 (3T3Am) cells and naïve 3T3 cells were used as target cells and SU positive foci were determined by MAb 697. As controls, viruses generated by CasE+4070A- and hrGFP+4070A-NSC culture were evaluated in parallel.

## Competing interests

The authors declare that they have no competing interests.

## Authors' contributions

YL carried out the majority of the experimental studies, helped design the experiments, analyzed the data and provided a preliminary draft of the manuscript. WPL conceived of the study, participated in its design and coordination, performed experiments, analyzed the data, and drafted the manuscript. Both authors read and approved the final manuscript.

## References

[B1] Gardner MB Retroviral spongiform polioencephalomyelopathy Rev Infect Dis 1985 7 99 110 2984760 10.1093/clinids/7.1.99

[B2] Gardner MB Henderson BE Officer JE Rongey RW Parker JC Oliver C Estes JD Huebner RJ A spontaneous lower motor neuron disease apparently caused by indigenous type-C RNA virus in wild mice JNCI 1973 51 1243 1254 4355605 10.1093/jnci/51.4.1243 PMC7204280

[B3] Gardner MB Rasheed S Klement V Rongey RW Brown JC Dworsky R Henderson BE Lower motor neuron disease in wild mice caused by indigenous type C virus and search for a similar etiology in human amyotrophic lateral sclerosis UCLA Forum Med Sci 1976 19 217 234 191966

[B4] DesGroseillers L Barrette M Jolicoeur P Physical Mapping of the paralysis-inducing determinant of a wild mouse ecotropic neurotropic virus J Virol 1984 52 356 363 6092665 10.1128/jvi.52.2.356-363.1984 PMC254534

[B5] Jolicoeur P Gravel C Kay DG Roos RP Pathogenesis of murine spongiform myeloencephalopathy induced by a murine retrovirus Molecular Neurovirology 1992 Totowa, NJ: Humana Press Inc 199 224

[B6] Portis JL Genetic determinants of neurovirulence of murine oncornaviruses Adv Virus Res 2001 56 3 38 10.1016/S0065-3527(01)56003-0 11450304

[B7] Portis JL Lynch WP Dissecting the determinants of neuropathogenesis of the murine oncornaviruses Virology 1998 247 127 136 10.1006/viro.1998.9240 9705905

[B8] Wong PKY Yuen PH Roos R Molecular basis of neurologic disorders induced by a mutant ts1, of Moloney murine leukemia virus Molecular Neurovirology: Pathogenesis of Viral CNS Infections 1992 Totowa: Humana Press 161 197

[B9] Murphy SL Honczarenko MJ Dugger NV Hoffman PM Gaulton GN Disparate regions of envelope protein regulate syncytium formation versus spongiform encephalopathy in neurological disease induced by murine leukemia virus TR J Virol 2004 78 8392 8399 10.1128/JVI.78.15.8392-8399.2004 15254211 PMC446142

[B10] Paquette Y Hanna Z Savard P Brousseau R Robitaille Y Jolicoeur P Retrovirus-induced murine motor neuron disease: Mapping the determinant of spongiform degeneration within the envelope gene Proc Natl Acad Sci USA 1989 86 3896 3900 10.1073/pnas.86.10.3896 2542954 PMC287248

[B11] Lynch WP Snyder EY Qualtiere L Portis JL Sharpe AH Late virus replication events in microglia are required for neurovirulent retrovirus-induced spongiform neurodegeneration: evidence from neural progenitor-derived chimeric mouse brains J Virol 1996 70 8896 8907 8971019 10.1128/jvi.70.12.8896-8907.1996 PMC190987

[B12] Kay DG Gravel C Pothier F Laperriere A Robitalle Y Jolicoeur P Neurological disease induced in transgenic mice expressing the env gene of the Cas-Br-E murine retrovirus Proc Natl Acad Sci USA 1993 90 4538 4542 10.1073/pnas.90.10.4538 8389454 PMC46547

[B13] Yu YE Choe W Zhang W Stoica G Wong PK Development of pathological lesions in the central nervoous system of transgenic mice expressing the env gene of ts1 Moloney murine leukemia virus in the absence of viral gag and pol genes and viral replication Journal of Neuorvirololgy 1997 3 274 282 10.3109/13550289709029468 9291235

[B14] Lynch WP Sharpe AH Snyder EY Neural stem cells as engraftable packaging lines can mediate gene delivery to microglia: evidence from studying retroviral env-related neurodegeneration J Virol 1999 73 6841 6851 10400782 10.1128/jvi.73.8.6841-6851.1999 PMC112769

[B15] Chesebro B Trifilo M Race R Meade-White K Teng C LaCasse R Raymond L Favara C Baron G Priola S Anchorless prion protein results in infectious amyloid disease without clinical scrapie Science 2005 308 1435 1439 10.1126/science.1110837 15933194

[B16] Albritton LM Tseng L Scadden D Cunningham JM A putative murine ecotropic retrovirus receptor gene encodes a multiple membrane-spanning protein and confers susceptibility to virus infection Cell 1989 57 659 666 10.1016/0092-8674(89)90134-7 2541919

[B17] Barnett AL Davey RA Cunningham JM Modular organization of the Friend murine leukemia virus envelope protein underlies the mechanism of infection PNAS 2001 98 4113 4118 10.1073/pnas.071432398 11274436 PMC31188

[B18] Snyder EY Deitcher DL Walsh C Arnold-Aldea S Hartwieg E Cepko CL Multipotent neural cell lines can engraft and participate in development of mouse cerebellum Cell 1992 68 33 51 10.1016/0092-8674(92)90204-P 1732063

[B19] Flynn JA An W King SR Telesnitsky A Nonrandom dimerization of murine leukemia virus genomic RNAs J Virol 2004 78 12129 12139 10.1128/JVI.78.22.12129-12139.2004 15507599 PMC525042

[B20] Davey RA Hamson CA Healey JJ Cunningham JM In vitro binding of purified murine ecotropic retrovirus envelope surface protein to its receptor, MCAT-1 J Virol 1997 71 8096 8102 9343158 10.1128/jvi.71.11.8096-8102.1997 PMC192264

[B21] Fass D Davey RA Hamson CA Kim PS Cunningham JM Berger JM Structure of a Murine Leukemia Virus Receptor-Binding Glycoprotein at 2.0 Angstrom Resolution Nature 1997 277 1662 1666 10.1126/science.277.5332.1662 9287219

[B22] Lavillette D Boson B Russell SJ Cosset FL Activation of membrane fusion by murine leukemia viruses is controlled in cis or in trans by interactions between the receptor-binding domain and a conserved disulfide loop of the carboxy terminus of the surface glycoprotein J Virol 2001 75 3685 3695 10.1128/JVI.75.8.3685-3695.2001 11264358 PMC114860

[B23] Anelli T Sitia R Protein quality control in the early secretory pathway Embo J 2008 27 315 327 10.1038/sj.emboj.7601974 18216874 PMC2234347

[B24] Helenius A Aebi M Roles of N-linked glycans in the endoplasmic reticulum Annu Rev Biochem 2004 73 1019 1049 10.1146/annurev.biochem.73.011303.073752 15189166

[B25] Vembar SS Brodsky JL One step at a time: endoplasmic reticulum-associated degradation Nat Rev Mol Cell Biol 2008 9 944 957 10.1038/nrm2546 19002207 PMC2654601

[B26] Gardner MB Type C viruses of wild mice-Characterization and natural history of amphotropic, ecotropic, and xenotropic MuLV Curr Top Microbiol Immunol 1978 79 215 259 206407 10.1007/978-3-642-66853-1_5

[B27] Perryman S Nishio J Chesebro B Complete nucleotide sequence of Friend murine leukemia virus, strain FB29 Nucleic Acids Res 1991 19 6950 10.1093/nar/19.24.6950 1762923 PMC329334

[B28] Portis JL Czub S Garon CF McAtee FJ Neurodegenerative disease induced by the Wild Mouse ecotropic retrovirus is markedly accelerated by long terminal repeat and *gag-pol *sequences from nondefective Friend murine leukemia virus J Virol 1990 64 1648 1656 2181155 10.1128/jvi.64.4.1648-1656.1990 PMC249301

[B29] Kozak SL Kabat D Ping-pong amplification of a retroviral vector achieves high level gene expression: human growth hormone production J Virol 1990 64 3500 3508 2352330 10.1128/jvi.64.7.3500-3508.1990 PMC249616

[B30] Ryder EF Snyder EY Cepko CL Establishment and characterization of multipotent neural cell lines using retrovirus vector-mediated oncogene transfer Jounal of Neurobiology 1990 21 356 375 10.1002/neu.480210209 2307979

[B31] Miller AD Law M-F Verma IM Generation of helper-free amphtropic retroviruses that transduce a dominant-acting, methotrexate-resistant dihydrofolate reductase gene Mol Cell Biol 1985 5 431 437 2985952 10.1128/mcb.5.3.431-437.1985 PMC366734

[B32] McAtee FJ Portis JL Monoclonal antibodies specific for wild mouse neurotropic retrovirus: Detection of comparable levels of virus replication in mouse strains susceptible and resistent to paralytic disease J Virol 1985 56 1010 1022 10.1128/jvi.56.3.1018-1022.1985 PMC252677 3877818

[B33] Lynch WP Brown WJ Spangrude GJ Portis JL Microglial infection by a neurovirulent murine retrovirus results in defective processing of envelope protein and intracellular budding of virus particles J Virol 1994 68 3401 3409 8151801 10.1128/jvi.68.5.3401-3409.1994 PMC236834

[B34] Czub M Czub S McAtee F Portis J Age-dependent resistance to murine retrovirus-induced spongiform neurodegeneration results from CNS-specific restriction of virus replication J Virol 1991 65 2539 2544 1850027 10.1128/jvi.65.5.2539-2544.1991 PMC240610

[B35] Evans LH Morrison RP Malik FG Portis J Britt WJ A neutralizable epitope common to the envelope glycoproteins of ecotropic, polytropic, xenotropic, and amphotropic murine leukemia viruses J Virol 1990 64 6176 6183 1700832 10.1128/jvi.64.12.6176-6183.1990 PMC248792

[B36] Czub M McAtee FJ Portis JL Murine retrovirus-induced spongiform encephalomyelopathy: host and viral factors which determine the length of the incubation period J Virol 1992 66 3298 3305 1316449 10.1128/jvi.66.6.3298-3305.1992 PMC241107

[B37] Miller AD Wolgamot G Murine retroviruses use at least six different receptors for entry into Mus dunni cells J Virol 1997 71 4531 4535 9151846 10.1128/jvi.71.6.4531-4535.1997 PMC191674

[B38] Chesebro B Britt W Evans L Wehrly K Nishio J Cloyd M Characterization of monoclonal antibodies reactive with murine leukemia viruses: use in analysis of strains of friend MCF and Friend ecotropic murine leukemia virus Virology 1983 127 134 148 10.1016/0042-6822(83)90378-1 6305011

[B39] Robertson MN Miyazawa M Mori S Caughey B Evans LH Hayes SF Chesebro B Production of monoclonal antibodies reactive with a denatured form of the Friend murine leukemia virus gp70 envelope protein: use in a focal infectivity assay, immunohistochemical studies, electron microscopy and western blotting J Virol Methods 1991 34 255 271 10.1016/0166-0934(91)90105-9 1744218

[B40] Lynch WP Sharpe AH Differential glycosylation of the Cas-Br-E env protein is associated with retrovirus-induced spongiform neurodegeneration J Virol 2000 74 1558 1565 10.1128/JVI.74.3.1558-1565.2000 10627570 PMC111494

